# A homozygous p.Glu150Lys mutation in the opsin gene of two Pakistani families with autosomal recessive retinitis pigmentosa

**Published:** 2009-12-03

**Authors:** Maleeha Azam, Muhammad Imran Khan, Andreas Gal, Alamdar Hussain, Syed Tahir Abbas Shah, Muhammad Shakil Khan, Ahmed Sadeque, Habib Bokhari, Rob W.J. Collin, Ulrike Orth, Maria M. van Genderen, A.I. den Hollander, Frans P. M. Cremers, Raheel Qamar

**Affiliations:** 1 Department of Biosciences, COMSATS Institute of Information Technology, Islamabad, Pakistan; 2 Department of Human Genetics, Radboud University Nijmegen Medical Centre, Nijmegen, The Netherlands; 3 Department of Human Genetics, University Medical Centre Hamburg Eppendorf, Germany; 4 Shifa College of Medicine, Islamabad, Pakistan; 5 Nijmegen Centre for Molecular Life Sciences, Radboud University Nijmegen Medical Centre, Nijmegen, The Netherlands; 6 Bartiméus Institute for the Visually Impaired, Zeist, The Netherlands

## Abstract

**Purpose:**

To identify the gene mutations responsible for autosomal recessive retinitis pigmentosa (arRP) in Pakistani families.

**Methods:**

A cohort of consanguineous families with typical RP phenotype in patients was screened by homozygosity mapping using microsatellite markers that mapped close to 21 known arRP genes and five arRP loci. Mutation analysis was performed by direct sequencing of the candidate gene.

**Results:**

In two families, RP21 and RP53, homozygosity mapping suggested *RHO,* the gene encoding rhodopsin, as a candidate disease gene on chromosome 3q21. In six out of seven affected members from the two families, direct sequencing of *RHO* identified a homozygous c.448G>A mutation resulting in the p.Glu150Lys amino acid change. This variant was first reported in PMK197, an Indian arRP family. Single nucleotide polymorphism analysis in RP21, RP53, and PMK197 showed a common disease-associated haplotype in the three families.

**Conclusions:**

In two consanguineous Pakistani families with typical arRP phenotype in the patients, we identified a disease-causing mutation (p.Glu150Lys) in the *RHO* gene. Single nucleotide polymorphism analysis suggests that the previously reported Indian family (PMK197) and the two Pakistani families studied here share the *RHO* p.Glu150Lys mutation due to a common ancestry.

## Introduction

Retinitis pigmentosa (RP) is a clinically and genetically heterogeneous group of ophthalmic diseases that are characterized by night blindness and gradual loss of peripheral vision due to a progressive degeneration of the photoreceptor cells in the retina. RP is a major cause of inherited blindness in adulthood, with a worldwide prevalence of 1 in 3,500–4,000 individuals [1-4]. The diagnosis of individuals affected by RP is typically based on fundus examination and the electroretinogram (ERG). In affected individuals, the fundus shows a waxy pallor of the optic discs, retinal vessel attenuation, and peripheral pigmentary alterations with bone spicule deposits. The ERG typically shows a photoreceptor dysfunction with the rod-derived responses being more affected than the cone-derived ones [5]. In addition to clinical heterogeneity of RP, there is a significant genetic heterogeneity of the disease, with different modes of inheritance [6-8]. To date more than 40 different RP-associated genes have been identified (RetNet). These genes encode proteins that are involved either in the phototransduction cascade, retinoid metabolism, cell–cell interaction, or photoreceptor development. Some of these proteins are transcription or splicing factors, whereas others are intracellular transport molecules [9,10].

Rhodopsin is an abundant rod photoreceptor-specific transmembrane protein, which upon photoexcitation initiates the phototransduction cascade. Heterozygous mutations of the corresponding gene (*RHO*; MIM 180380) account for approximately 30% of all autosomal dominant RP (adRP) cases in the USA and the UK, whereas the relative frequency and prevalence of *RHO* mutations is uncertain in the Asian population [11,12]. Up to now there have been more than 100 adRP-causing mutations, but only two autosomal recessive RP-associated mutations have been reported in the coding sequence of *RHO* (RetNet) [13-15]. The first arRP case, a French-Canadian female patient, carried an apparently homozygous null mutation in exon 4 [14], whereas in the second case, in a consanguineous Indian family, a homozygous c.448G>A (p.Glu150Lys) mutation in exon 2 was reported [15]. Here we report the identification of the p.Glu150Lys *RHO* mutation in affected individuals from two Pakistani families with arRP.

## Methods

### Family collection

A total of 29 arRP families representative of the autosomal recessive form of the disease were selected from different areas of northern and central Pakistan.

### Pedigree analysis and clinical ophthalmic examination

**Figure 1:**
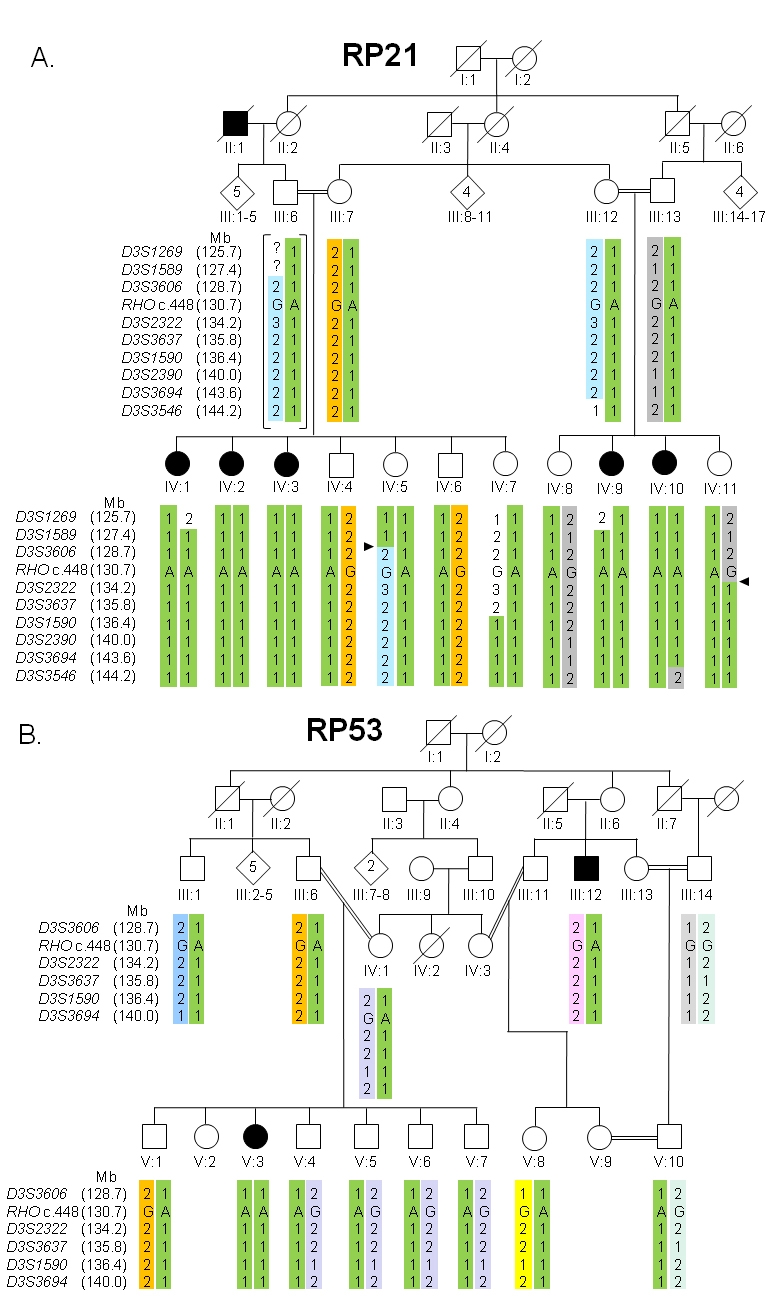
Pedigrees and 3q21 marker haplotypes spanning the *RHO* gene in autosomal recessive retinitis pigmentosa families RP21 (**A**) and RP53 (**B**). **A**: Family RP21 consists of four generations. White circles represent unaffected females, filled circles affected females, white squares unaffected males, and filled squares affected males. Deceased individuals are shown with a slanting line across the symbol. Also shown are the marker loci and their positions in an 18.5-Mb interval, which were used for fine mapping of the disease locus. Marker allele sizes were not accurately determined and therefore we only give two-allele designations. Arrowheads designate crossovers that define the 6.8-Mb minimal critical region between *D3S1589* and *D3S2322*. **B**: Family RP53 consists of five generations. Patient V-3 in one branch of the family is homozygous for marker alleles flanking *RHO*. The disease in affected individual III-12 could be *RHO* unrelated, suggesting locus heterogeneity in the different branches of the family. Haplotypes were established for all individuals for which marker alleles are depicted, except for RP21-III-6, the haplotypes of whom were deduced (indicated with brackets around the haplotypes).

After an initial linkage analysis, described below, two consanguineous Pakistani families were ascertained; RP21 was from Dera Ghazi Khan (central Pakistan) and family RP53, which was identified later during control panel screening for the *RHO* mutation, was from Abbottabad (northern Pakistan). Affected individuals had typical RP symptoms with early onset of night blindness. Medical and family histories of the participants were then obtained with the help of a questionnaire, and the pedigree structure was drawn (Figure 1) [16]. Clinical examination and characterization of the affected and unaffected individuals was done by an experienced ophthalmologist. For this purpose, individuals IV-1 and IV-7 of RP21 and V-1 and V-3 of RP53 were then selected for further detailed investigations, including funduscopy of the retina and ERG studies.

### Ethics committee approval

This study adhered to the tenets of the Declaration of Helsinki. The consent of both families was obtained for this study, which was approved by the Shifa College of Medicine, Shifa International Hospital Ethics Committee Institutional Review Board after an extensive review of the protocol.

### Sample collection and DNA isolation

Peripheral blood samples of each individual, for which haplotypes are depicted in Figure 1, except RP21-III-6 were drawn by 10 ml sterile syringes (Terumo Co. BL, Philipines) and were collected using vacutainer tubes containing acid citrate dextrose (Becton Dickinson, Franklin Lakes, NJ). All the blood samples were stored at 4 °C till processed further. Blood samples of 100 random, unrelated, healthy control individuals were also collected and stored as above for determination of allele frequency in the general Pakistani population. Genomic DNA was isolated using an organic phenol-chloroform extraction method [17,18], briefly the protocol consisted of cell lysis and protein digestion followed by phenol-chloroform extraction of the peptides, and precipitation of DNA with ethanol. The precipitated DNA was re-suspended in Tris-EDTA buffer and stored at 4 °C before use.

### Linkage mapping and haplotype analysis of arRP genes

To find the causative gene in the arRP families, homozygosity mapping using polymorphic microsatellite markers for the known arRP loci was performed. Microsatellite markers mapped by the Cooperative Human Linkage Center were obtained from Invitrogen, Inc. (Carlsbad, CA). Cytogenetic locations of these markers as well as the length of the amplified products were obtained from the Human Genome Database and the Marshfield Medical Center database. Table 1 lists the arRP genes and loci with the flanking markers used for homozygosity mapping. Each microsatellite marker was amplified in a 25-μl volume consisting of the following: 0.5 mM deoxynucleotide triphosphates (dNTPs), 1X Taq Buffer (10 mM Tris-HCl, pH 9.0, 50 mM KCl, 0.1% Triton X-100, 0.01% [w/v] gelatin), 1.5 mM MgCl_2_, 0.3 μM of each forward and reverse primer, 2.5 U Taq polymerase, and 50 ng genomic DNA. The thermal cycling conditions were as follows: initial denaturation of 90 °C for 2 min, followed by 40 cycles of 90 °C for 1 min, 55 °C for 1 min, 72 °C for 1 min, and a final extension at 72 °C for 7 min. Genotypes were defined following PCR and 8% PAGE. Images of the gels were visualized by UV transillumination after staining with ethidium bromide, captured with a digital camera, and documented using the software BioCap MW Version 11.01 software from Vilber Lourmat (Marne-La-Vallee Cedex1, France). Haplotypes were constructed based upon the allelic patterns of microsatellites.

**Table 1:** Microsatellite marker alleles flanking known genes or loci for arRP in selected individuals of families RP21 and RP53.

**Loci**	**Markers**	**cM**	**RP21**				**RP53**			
			**Parent** **III-12**	**Parent** **III-13**	**Affected** **IV-9**	**Affected** **IV-10**	**Parent** **III-6**	**Parent** **IV-1**	**Affected** **V-3**	**Affected** **III-12**
**ABCA4**	D1S406	125.5	1,2	1,2	1,2	1,2	1,2	1,2	1,2	1,2
	D1S1587	126.2	1,2	1,2	1,2	1,2	1,2	1,2	1,2	1,2
**CRB1**	D1S2816	211.1	1,1	1,1	1,1	1,1	1,1	1,1	1,1	1,1
	D1S1660	212.4	1,2	1,1	2,1	2,1	1,2	1,2	1,2	1,2
**USH2A**	D1S474	233.9	1,2	1,2	1,2	1,2	1,2	1,2	1,2	1,2
	D1S2827	234.5	1,2	1,2	1,2	1,2	1,2	1,2	1,2	1,2
**RPE65**	D1S2806	100.4	1,2	1,3	2,1	2,1	1,1	1,1	1,1	1,1
	D1S219	101.5	1,2	1,2	2,1	2,1	2,1	1,3	1,1	2,1
**RP32**	D1S2808	131.9	1,2	1,1	2,1	1,1	1,2	3,1	1,1	1,2
	D1S2896	134.2	1,1	2,1	1,1	1,1	1,1	1,1	1,1	1,1
	D1S485	136.9	1,2	1,2	2,1	1,2	1,2	1,2	1,2	1,2
**SAG**	D2S2348	242.2	2,1	1,2	1,2	1,1	1,1	1,2	1,2	1,2
	D2S2205	243	1,2	1,2	1,2	1,2	1,2	1,2	1,2	1,2
**CERKL**	D2S2261	185.1	2,2	1,1	1,2	1,2	1,2	1,2	1,2	1,1
	D2S1391	186.2	1,2	1,1	1,2	1,1	1,1	1,2	1,2	1,2
**MERTK**	D2S1888	121.6	1,1	1,1	1,1	1,1	1,1	1,1	1,1	1,1
	D2S1892	122.1	1,2	1,3	1,3	1,3	1,2	1,1	1,2	1,2
**RP28**	D2S2225	82.8	1,1	1,1	1,1	1,1	1,1	1,2	1,2	1,1
	D2S1337	85	1,2	1,2	1,2	1,2	1,2	1,2	1,2	1,2
**RHO**	D3S3607	143.9	1,1	1,2	1,1	1,1	1,2	1,2	1,1	1,1
	D3S2322	146.6	1,2	1,2	1,1	1,1	1,2	1,2	1,1	1,2
	D3S1587	146.6	1,3	1,2	1,1	1,1	1,2	1,2	1,1	1,2
**LRAT**	D4S1586	147	1,2	1,2	1,2	1,2	1,2	1,2	1,2	1,2
	D4S2962	152.9	1,2	1,2	1,2	1,2	1,2	1,2	1,2	1,2
**CNGA1**	D4S1627	60.1	1,2	1,1	1,1	1,1	1,2	1,3	1,3	1,2
	D4S3255	61.4	2,2	2,2	2,2	2,2	1,2	1,2	1,1	1,1
**PDE6B**	D4S3360	0	1,1	1,1	1,1	1,1	1,1	1,1	1,1	1,1
	D4S2936	1.4	1,3	1,3	1,3	1,3	1,2	1,2	1,2	1,2
**RP29**	D4S621	175.6	1,1	1,1	1,1	1,1	1,1	1,1	1,1	1,1
	D4S415	181.3	1,1	1,1	1,1	1,1	1,1	1,2	1,1	1,2
**PROM1**	D4S403	25.9	1,1	1,1	1,1	1,1	1,2	1,2	1,1	1,2
	D4S3048	29.1	1,2	1,3	1,3	1,3	1,3	1,1	1,3	1,2
**PDE6A**	D5S812	150.3	1,1	1,1	1,1	1,1	1,3	1,2	1,2	1,2
	D5S2013	152.6	1,2	1,1	1,1	1,1	1,2	1,2	1,2	1,2
**TULP1**	D6S265	44.4	1,2	1,2	1,2	2,1	1,2	1,3	1,2	1,3
	D6S1645	48.3	1,2	1,1	1,1	2,1	1,2	1,2	1,2	1,2
**RP25**	D6S257	79.9	1,2	1,2	2,1	2,1	1,2	1,2	2,1	1,2
	D6S1644	96.1	1,2	1,1	2,1	2,1	1,1	1,2	1,1	1,2
**RP1**	D8S509	69.4	1,2	1,1	1,1	2,1	1,2	1,2	1,2	1,2
	D8S1828	71	1,1	1,3	1,3	1,1	1,1	1,3	1,3	1,2
**RGR**	D10S219	100.9	1,2	1,1	1,1	1,2	1,2	1,1	2,1	1,2
	D10S523	103.4	1,1	1,2	1,2	2,1	1,1	1,3	1,3	1,2
**NRL**	D14S50	12.5	1,2	1,3	1,2	1,1	1,2	1,2	2,1	1,1
	D14S283	14	1,1	1,2	1,1	1,1	1,1	1,2	1,1	1,1
**RLBP1**	D15S116	85.6	1,2	1,2	1,1	2,1	1,2	1,2	1,2	1,2
	D15S996	86.8	1,2	1,3	2,1	2,1	1,2	1,1	1,1	1,2
**NR2E3**	D15S650	70.7	1,1	1,1	1,1	1,1	1,2	1,1	1,1	1,1
	D15S204	71.8	1,2	1,3	1,3	1,3	1,2	1,2	1,1	1,2
**CNGB1**	D16S3057	77.1	1,2	1,2	1,2	1,2	1,2	1,3	1,2	1,2
	D16S670	78.7	1,1	1,1	1,1	1,1	1,2	1,2	1,2	1,2
**RP22**	D16S403	43.8	1,3	1,2	3,1	1,2	1,1	1,2	1,2	1,2
	D16S287	48.6	1,1	1,1	1,1	1,1	1,2	1,2	1,2	1,3
**PRCD**	D17S1848	95.9	1,1	1,2	1,2	1,1	1,2	1,2	1,1	1,2
	D17S1839	102.5	1,2	1,2	1,1	2,1	1,2	1,2	1,2	1,2

After suggestive linkage was observed, additional flanking markers were selected from the above mentioned databases, and haplotypes were constructed for two generations in both the pedigrees. The Lod score analysis of all the genotyped individuals for family RP21 (excluding RP21-III-6 for whom the haplotype was logically deduced) for the markers was performed with the EasyLinkage software package version 5.02 (Friedrich Alexander University Erlangen, Nuremberg, Germany) [19].

### Sequence analysis of *RHO*

The sequencing primers for *RHO* were designed for the upstream promoter sequence and the five coding exons with Primer3 software (Whitehead Institute for Biomedical Research, Cambridge, MA; Table 2). All exons of the *RHO* gene were bidirectionally sequenced with an automated DNA sequencer.

**Table 2:** Amplification and sequencing primers for *RHO*

**Name**	**Forward primer (5′→3′)**	**Reverse Primer (5′→3′)**
Promoter 1	CTAGCGTTCAAGACCCATTAC	ACTCAGGATCCAGGAAAAGG
Promoter 1	GGACTGGATGACTCCAGAGG	CCAAGAATGCTGCGAAGG
Exon 1	CTGCAGCGGGGATTAATATG	GGACAGGAGAAGGGAGAAGG
Exon 2	GTTGCCTTCCTAGCTACCCTCT	GCCAGGAGACATACAAGGTCAG
Exon 3	CAGCCATGCAGACGTTTATG	GTCCAGACCATGGCTCCTC
Exon 4	ACGGCTCTGAGGGTCCAG	AGGAATCTGCATTTCTCACACA
Exon 5a	GAATCGTGAGGGGCAGAAG	AAAGATGAGTTGGGAGGAGGA
Exon 5b	GGGACATCCACCAAGACCTA	AAAATGTTCACTATCAGGAGGTGAT
Exon 5c	GGGCCTCACTTTCTTCTCCT	ATAGGCCACATTGGGAAGG
Exon 5d	AACCTTGGGGCAGGTTTTTA	CTTGTCTGGCAAGGGAAACT
Exon 5e	TCTCGAAGAGCTTAGAAACAAAGA	CCCAGCCAAGGTCAGTTTTA

### Statistical analysis

Genotype frequencies of cases and controls were compared to determine association/correlation, and chi-square (χ^2^) tests were performed at the 95% confidence interval to calculate the difference between the observed and the expected frequencies of the variant.

### Founder effect detection

**Figure 2:**
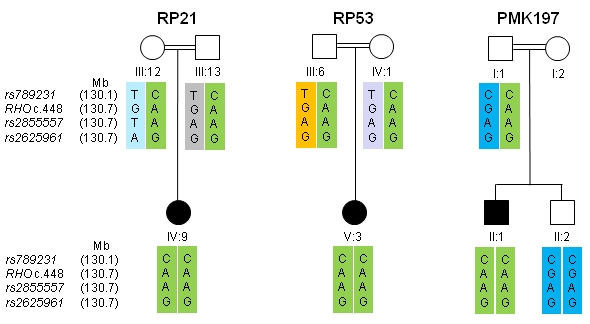
Haplotype analysis for single nucleotide polymorphisms (SNPs) in close proximity to the *RHO* mutation. Frequencies [26] of the combined homozygous variants based on individual allele frequencies in the studied population are: rs789231 (CC), 0.25; *RHO* (AA), 0.000025; rs2855557 (AA), 0.178084; rs2625961 (GG), 0.207025.

To determine the status of any founder effect in the Pakistani families RP21 and RP53 and the Indian family PMK197, microsatellite and single nucleotide polymorphism (SNP) analysis were performed in all three families. Informative SNPs surrounding the *RHO* gene were selected, using the UCSC genome browser (University of California Santa Cruz, Santa Clara, CA) and haplotypes were constructed for all three families (Figure 2).

## Results

**Figure 3:**
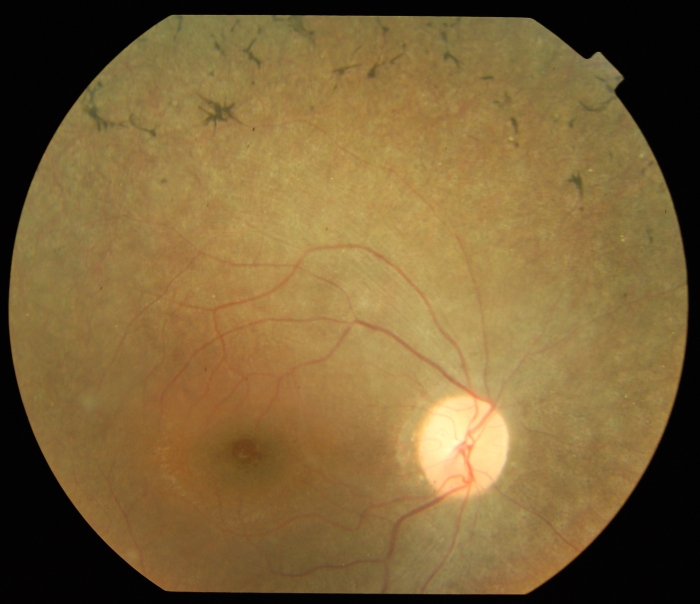
Fundus photograph of an individual affected with retinitis pigmentosa. The fundus appearance of individual RP21-IV-1 is characteristic of advanced RP, with optic disc pallor, attenuation of the retinal vessels, peripheral pigment epithelial atrophy, and bone spicule pigmentation. The posterior pole shows macular edema, and small white dots are present in the mid-periphery at the level of the retinal pigment epithelium

In family RP21, there are four generations with five affected individuals in the fourth generation (Figure 1A), suggesting that RP has an autosomal recessive inheritance pattern (Figure 1). The onset of RP in this family was in the first decade, with typical symptoms of the disease including night blindness and progressive visual field loss. Fundus examination of the affected members of the family revealed typical features of RP, including optic disc pallor, attenuation of the retinal vessels, atrophy of the peripheral retinal pigment epithelium (RPE), and formation of bone spicule pigmentation. In addition, affected individuals had macular edema in the posterior pole and small white dots in the mid-periphery at the level of the RPE (Figure 3). The ERG showed unrecordable rod and cone responses with undetectable 30-Hz flicker ERG (data not shown). In contrast, in unaffected individuals the fundus was completely normal and the rod response and the mixed rod–cone response had normal amplitudes and implicit times, with normal oscillatory potentials (data not shown). Single flash photopic ERG and photopic 30-Hz flicker responses were also normal, thus the ERG pattern was unremarkable in these individuals.

Homozygosity mapping by microsatellite marker analysis for family RP21 excluded all reported loci for arRP but *RHO* (Table 1), for which homozygosity was seen in all five affected individuals for all flanking markers. The flanking markers of the *RHO* locus used in our study were those described previously by Young et al. [20]. Refinement of the marker analysis (Figure 1A) resulted in a 6.8-Mb (4.8-cM) critical interval flanked by *D3S1589* and *D3S2322,* with a maximum Lod score, for all the genotyped individuals only, of 2.6 for marker *D3S3606* at a θ value of 0.00.

**Figure 4:**
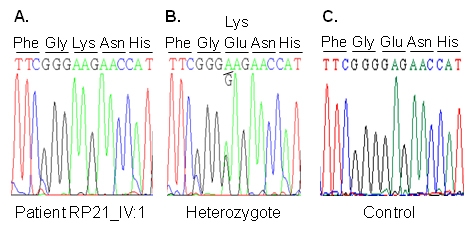
Sequence analysis of *RHO* in family RP21. **A**: Sequence trace of part of exon 2 of an affected individual (RP21-IV-1) showing the homozygous mutant sequence c.448G>A. **B**: Sequencing results of RP21-IV-7 showing the heterozygous c.448G>A sequence. **C**: Sequence trace of an unaffected individual with the homozygous wild-type c.448G. d

Direct sequencing of *RHO* resulted in identification of c.448G>A, a previously reported mutation in exon 2, resulting in the replacement of a lysine residue for a glutamic acid (p.Glu150Lys) [15]. All affected individuals in this family were found to be homozygous for this change, whereas all unaffected individuals were found to be heterozygous for this mutation (Figure 1 and Figure 4).

To determine the frequency of the c.448G>A mutation in the general Pakistani population, exon 2 of 100 random control samples was sequenced, which resulted in a frequency of 0.005 of the disease allele in the general population. During this screening of the unrelated healthy control panel, one individual (V-1, Figure 1B) was identified as a heterozygote of this mutation. Upon further investigation it was found that a person with RP was also present in this family. Detailed clinical examination of this family (RP53) confirmed the presence of RP, with onset of night blindness in the second decade of life in the two affected individuals. Blood samples of selected family members were then obtained and homozygosity mapping was conducted for the *RHO* locus (Figure 1B). Homozygosity mapping and sequencing resulted in identification of 11 heterozygotes, including the affected individual III-12, while only one female, V-3, was homozygous for the c.448G>A mutation. Sequencing of the remaining exons and upstream regulatory regions of *RHO* in III-12 did not reveal any additional *RHO* mutations.

In addition to the 100 control samples, exon 2 of the *RHO* gene was also sequenced using the DNA samples of 28 probands from a Pakistani RP cohort to determine the prevalence of the c.448G>A mutation. None of the affected individuals were found to carry this mutation. The χ^2^ analysis resulted in a significant association of the homozygous mutant allele A (p<0.05) with the disease when calculated for all the individuals of both the families and also when compared with the control samples (Table 3).

**Table 3:** Chi square analysis.

**A**	**Unaffected family members**	**Patients**	**p (χ^2^)**
GG/GA	20	1	<0.05 (22.04)
AA	0	6	
**B**	**Controls+Unaffected family members**	**Patients**	**p (χ^2^)**
GG/GA	119	1	<0.05 (107.10)
AA	0	6	

**A**: χ^2^ analysis of families RP21 and RP53 showing association of alleles AA with the disease. **B**: χ^2^ analysis of the families and the controls.

Founder effect analysis of the c.448G>A mutation in the two Pakistani families (RP21, RP53) and the Indian family (PMK197) [15] revealed that all affected members share the same homozygous haplotype consisting of three highly informative SNPs surrounding the *RHO* gene mutation (Figure 2), therefore suggesting a common ancestry between the three families.

## Discussion

Two apparently unrelated, ethnically diverse, Pakistani arRP families from different geographical regions of Pakistan, RP21 from the Punjab province and RP53 from the North West Frontier province, were identified and analyzed by homozygosity mapping using highly informative microsatellite markers flanking the known arRP genes and loci. Initially, one of the families (RP21) showed homozygosity for the *RHO* (RP4) locus at 3q22.1 in all affected individuals of the family.

Mutations in the *rhodopsin* gene have previously been shown to result in RP. However, most of the *RHO* mutations have been found in families with adRP, Human Genome Mutation Database (HGMD, rhodopsin mutation), whereas only two mutations have been shown to cause RP with an autosomal recessive mode of inheritance [14,15].

In this study, we identified the c.448G>A mutation in the *RHO* gene in two different consanguineous arRP families from Pakistan and also determined the frequency of the pathogenic allele in the normal population. While determining the population frequency, we found the c.448G>A mutation in the heterozygous state in one out of 100 control samples, but we did not find it in any of the other 28 families from our RP panel that were screened for the c.448G>A mutation. Further investigations of the family of the heterozygous individual from the control samples resulted in the identification of an additional arRP family (RP53). DNA sequencing revealed the same causative mutation in one branch of the family. There seems to be genetic heterogeneity of the RP in this family as individual III-12 may either carry a second, yet unidentified, variant in the *RHO* intronic sequences or a mutation in a different gene. The statistical analysis for the association/correlation showed that the mutant allele A is significantly associated (p<0.05) with the disease phenotype because the AA genotype is always observed in known affected individuals and is absent from the normal population (Table 3, Figure 2).

This c.448G>A mutation has previously been reported to cause arRP in an Indian family from southern India [15], which is ethnically different and geographically isolated from the present families. We therefore compared, in the two Pakistani families and the Indian family, some of the SNPs from the surrounding region of *RHO* to determine if they share a haplotype, suggesting a founder mutation in these families. Indeed, our analysis revealed a shared haplotype in the patients of the three families, suggesting a common ancestry. This is remarkable as the three families belong to diverse ethnic groups and have lived at their present location for several generations with no relative having moved to any other part of the subcontinent, at least for the last 10 generations; thus any commonality in ancestry must be more than several hundred years old.

Genetic screening of RP families is not only helpful in understanding the molecular mechanism of the disease; it is also important for genetic counseling. Traditional consanguineous marriages are major risk factors for autosomal recessive diseases, including retinopathies. Families have little knowledge of the inheritance of the diseases, and through such studies as the present one, many families could be counseled appropriately, as was done for members of families RP21 and RP53.

The rod visual pigment rhodopsin consists of the apoprotein opsin and the covalently bound chromophore 11-*cis*-retinal [21]. This protein is a heptahelical G protein-coupled receptor synthesized at a high level in the rod inner segment and transported subsequently to the rod outer segment [22]. The seven transmembrane segments (H1–H7) are sequentially linked by extracellular (E1–E3) and cytoplasmic (C1–C3) loops [23]. The first reported null mutation (p.Glu249Ter) associated with arRP was shown to abolish the function of the helices H6 and H7, which contains the retinal binding site. Remarkably, this mutation caused an abnormal ERG pattern even in heterozygotes [14].

Residue Glu150, which is embedded at the edge of the phospholipid bilayer, is located near the C-terminal region of the C2 loop, which is important for proper folding of opsin and its binding to transducin and thus for the initiation of the phototransduction cascade [24]. However, Zhu et al. [25] have shown that the homozygous p.Glu150Lys change does not affect binding of opsin to transducin but instead affects the export of the protein opsin from the Golgi apparatus. This is due to improper glycosylation of the protein, which results in retention of the protein inside the Golgi, leading to an insufficient supply of opsin to the rod outer segments. The processing of rhodopsin protein has been shown to be normal in the p.Glu150Lys heterozygotes [25].

We conclude that linkage and mutation analysis of known retinal disease genes in Pakistani patients with RP resulted in the identification of a *RHO* mutation in two out of 30 families that very likely have a common ancestor and are also distantly related with the Indian family previously found to harbor this mutation. We also observed genetic heterogeneity in one of these two families. These findings have facilitated accurate genetic counseling of patients and unaffected family members.
